# Swallowing sounds in speech therapy practice: a critical analysis of the literature

**DOI:** 10.1590/S1679-45082013000400024

**Published:** 2013

**Authors:** Juliana Lopes Ferrucci, Laura Davison Mangilli, Fernanda Chiarion Sassi, Suelly Cecilia Olivan Limongi, Claudia Regina Furquim de Andrade

**Affiliations:** 1 Universidade de São Paulo, São Paulo, SP, Brazil.; 2 Hospital das Clínicas, Faculdade de Medicina, Universidade de São Paulo, São Paulo, SP, Brazil.

**Keywords:** Deglutition, Deglutition disorders, Auscultation, Evaluation

## Abstract

This study aimed to investigate international scientific papers published on the subject of cervical auscultation and its use in speech therapy. The study involved a qualitative review of the literature spanning the last 10 years. Articles were selected from the PubMed database using the following keywords: cervical auscultation, swallowing and swallowing disorders. Research was included that was conducted on adult humans (over 18 years of age) and was written in English. Each citation retrieved from the database was analyzed independently by each of the study researchers to ascertain its relevance for inclusion in the study. The methodology involved formulating the research question, locating and selecting studies and critically evaluating the articles according to the precepts of the Cochrane Handbook. As a result, 35 studies were identified; 13 articles were analyzed because they allowed access to the full text and were related directly to the subject. We found that the studies were performed with groups of healthy subjects and subjects with different types of base pathology. Some studies compared the patterns found in the different groups. Some of the research sought to study the pattern of swallowing sounds with different factors - evaluator experience, the specificity and sensitivity of the method and how to improve the technique of cervical auscultation through the use of instruments other than the stethoscope. The conclusion of this critical analysis is that cervical auscultation is an important tool to be used in conjunction with other assessment methods in the routine clinical evaluation of swallowing.

## RESUMO

Este estudo objetivou pesquisar artigos científicos internacionais publicados sobre a ausculta cervical e sua utilização na prática fonoaudiológica. Trata-se de estudo de revisão qualitativa da literatura, envolvendo os últimos 10 anos. Os artigos foram selecionados da base de dados PubMed, utilizando os descritores: *auscultation cervical*, *deglutition* e *deglutition disorders*. A pesquisa deveria ter sido realizada com seres humanos, adultos (maiores que 18 anos) e escrita no idioma inglês. Cada citação recuperada no banco de dados foi analisada independentemente pelos pesquisadores do estudo, visando à pertinência de sua seleção e de sua inclusão ou não no estudo. A metodologia empregada envolveu a formulação da pergunta, a localização e seleção dos estudos, e a avaliação crítica dos artigos, conforme os preceitos do *Cochrane Handbook*. Como resultados, foram identificados 35 estudos, dos quais 13 foram analisados, pois permitiram acesso ao texto completo e relacionavam-se diretamente ao tema. Concluiu-se que os estudos foram realizados com grupos de sujeitos saudáveis e sujeitos com diferentes tipos de patologia de base. Alguns estudos compararam os padrões encontrados nos diferentes grupos. Constatou-se que parte dos artigos buscou estudar o padrão dos sons da deglutição, com diferentes interfaces: a experiência dos avaliadores; especificidade e sensibilidade do método; e como aprimorar a técnica da ausculta cervical por meio da utilização de outros instrumentos, diferentes do estetoscópio. Com essa análise crítica, concluiu-se que utilização da ausculta cervical é importante como um instrumento utilizado juntamente de outros, na rotina da avaliação clínica da deglutição.

## INTRODUCTION

Cervical auscultation (CA) is a clinical method used to evaluate the pharyngeal phase of swallowing. This method is a non-invasive procedure that is easy to perform and inexpensive^(1)^. It consists of placing a stethoscope on the lateral border of the trachea above the cricoid cartilage^(2)^ so that the stethoscope amplifies the sounds of swallowing and breathing. Sounds suggesting normal or impaired swallowing can be analyzed and interpreted by the listener^(3)^. With this method, it is possible to investigate laryngeal penetration and aspiration^(4)^. Therefore, CA is a useful clinical tool for the early identification of patients at high risk for penetration/aspiration^(1,5)^.

The use of this method alone to evaluate swallowing is still not accepted unanimously among researchers. The literature has discussed factors such as the subjectivity of CA^(2,6)^, interobserver variability^(7)^ and the need for training to distinguish the various sounds of the cervical region^(6,8)^.

Recent studies^(1,6,9)^ have advocated the use of CA as a complementary tool in the clinical evaluation of dysphagia. The authors state that CA is a promising method because it is not invasive, and its use in conjunction with clinical evaluation helps speech therapists to achieve better results^(1)^. However, most authors agree that more studies are needed before the use of this technique can be validated.

## OBJECTIVE

The purpose of this literature review was to gather information from international scientific texts published on CA and its use in speech therapy.

## METHODS

The principles of the Cochrane Handbook^(10)^ were used to establish the method. A survey was conducted of scientific texts on CA published in the last 10 years. The articles were selected from the PubMed database using the following keywords: cervical auscultation, swallowing, and swallowing disorders. The search was limited to research with adult humans written in the English language.

Searches for texts in the database were independently performed by the authors, minimizing the possibility of missing citations. Each citation was independently analyzed by each researcher to ascertain its relevance for inclusion in the study. In the event of disagreement, only texts agreed on by all of the researchers were included in the final selection.

Citations in languages other than English were excluded, as were citations that did not allow access to the full text (CAPES Portal) or that were repeated due to overlapping keywords. Case studies, literature reviews, letters to the editor and texts that were not directly related to the subject (*e.g.*, pulmonary auscultation, esophageal sounds) were also excluded. Only papers that were related to the proposed study were analyzed.

## RESULTS

**Figure 1:**
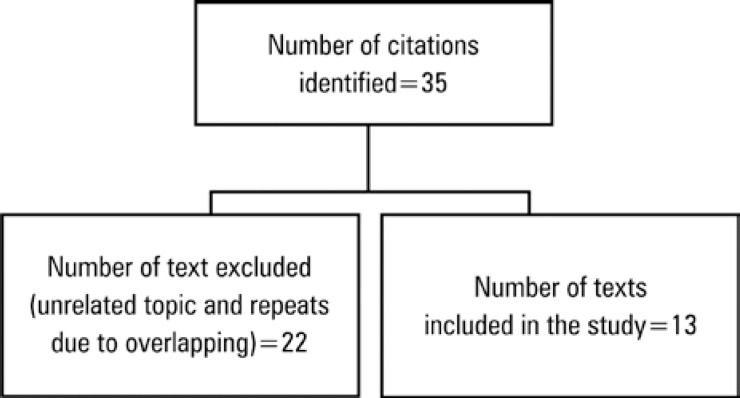
Search routine for the selection of texts to be analyzed

The search routine for selecting texts is shown in figure 1.

After the search and article selection, the texts were analyzed to ascertain the number, gender and age of the participants, their evaluation criteria and the results.

The main findings of each of the selected texts were then established as follows.

Researchers^(10)^ conducted a study to develop a new technique for the evaluation of swallowing. The authors evaluated 60 healthy and 15 dysphagic subjects using a microphone attached to the lateral border of the trachea to amplify and record the sounds. The healthy group was offered oral intake of solid, semi-solid, semi-liquid and liquid consistencies. The dysphagic patients were only offered 10mL of water. The parameter used for the analysis was the mean duration of swallowing sounds. Videofluoroscopy was performed simultaneously with the recording to check for penetration/aspiration. The results indicated that the mean duration of the sounds for the liquid bolus was significantly higher in dysphagic patients compared with controls. A sensitivity of 0.67 and a specificity of 1.0 for the penetration/aspiration aspect were found when comparing the tests. The authors concluded that the proposed technique could be incorporated into the clinical evaluation but should not replace other, more valuable diagnostic measures.

Another study^(11)^ used acoustic analysis to verify the possibility of determining patterns of normality. A microphone was used to capture and record swallowing sounds in 59 healthy subjects. For the evaluation, the subjects were offered juice in volumes of 5mL, 10mL and 15mL (two offerings of each), ending the sample with the greatest swallowing volume. Acoustic analysis was performed to determine the patterns of the duration, intensity and frequency. The results indicated that the mean duration was 0.4 seconds, the mean intensity was 43dB, and mean frequency was 2200Hz. The study also demonstrated that normal swallowing was sensitive to aging and the volume of the bolus ingested.

One study^(5)^ compared acoustic and temporal measurements of swallowing in relation to consistency, age and gender for 97 normal adults. An accelerometer was positioned at the midline of the cricoid cartilage, and the subjects were offered an intake of puree, honey, thin liquid and solid consistencies. The results showed that the pharyngeal transit time was shorter for the less viscous food. Increasing age was correlated with longer swallowing duration. The mean swallowing time was 530ms, with a peak intensity of approximately 60dB and a peak frequency of 2304Hz. The authors noted that their results could serve as a basis for comparison for swallowing to provide a better understanding of its physiology.

One study^(3)^ evaluated the correlation between sounds and the physiological events of swallowing. Deglutition sounds were recorded by means of a stethoscope, and laryngoscopy was performed simultaneously. Twenty healthy adults were evaluated and offered 5 and 20mL of water and 5mL of yogurt. The results revealed that no acoustic component could be clearly identified under any particular condition. However, comparison of the swallowing sounds and events suggested associations between a pre-click and the onset of apnea, a pre-click and the start of epiglottis excursion, a click and the epiglottis returning to rest and a click and the end of the swallowing apnea. The authors noted that it was not possible to demonstrate a significant correlation between the sounds and the physiological events of swallowing.

A study^(12)^ verifying the existence of differences between swallowing sounds was conducted with two groups: a control group, consisting of 12 healthy children and 3 healthy adults, and a swallowing disorders group, consisting of 11 adults. For the evaluation, the subjects were offered thin and thick liquids and semisolids. The sounds were recorded using an accelerometer. A final screening algorithm correctly identified the 13 control subjects and the 11 subjects with swallowing disorders. The authors concluded that the proposed method was able to assist in the evaluation of swallowing sounds, reducing the need for objective examination.

Another study^(13)^ investigated the signals of swallowing sounds and selected the most important and discriminative features. Six healthy children and 2 healthy adults and 6 adults with dysphagia were analyzed. The subjects were offered 5mL of thickened liquid. The sounds were recorded by an accelerometer accompanied by simultaneous videofluoroscopy data. The results indicated that low and high frequency components represented the principal characteristics of the initial swallowing sounds, whereas middle frequency components characterized the sounds made during bolus passage.

Sixteen adult dysphagic patients were evaluated to assess inter- and intraobserver reliability during CA^(7)^. A stethoscope, microphone and video recorder were positioned on the thyroid cartilage, and the patient underwent simultaneous videofluoroscopy. Five speech therapists were instructed to check for the possible presence of aspiration in the sound recordings, which were evaluated at two different times in a random order and blinded to the images. There was a high level of agreement regarding the occurrence of aspiration, although a high rate of false positives was observed during swallowing when there was no aspiration. The authors argued that the speech therapists who achieved high reliabilities used certain internal criteria to interpret the sounds, emphasizing that more qualitative research is needed to ascertain the criteria adopted.

Other researchers^(14)^ performed a study to identify the origin of the components of swallowing sounds by simultaneously analyzing acoustic and radiological data. Fifteen healthy adults were evaluated. The recordings were made using a microphone positioned at the cricoid cartilage, and the subjects were offered 10mL of barium sulfate. The results were used to determine the origin of the three main component sounds of swallowing, relating them to the movements of anatomical structures and the different positions of the bolus: the sound of the rise of the larynx corresponded to the moment when the bolus was in the oropharynx/hypopharynx; the sound of the opening of the upper sphincter corresponded to the transit of the bolus through that region; and the sound of the descent and opening of the larynx corresponded to the presence of the bolus in the esophagus. The authors concluded that the sound of the upper sphincter opening was present in all swallowing and that this should be considered the reference component for the other events.

Another study^(6)^ sought to establish whether the interpretation of CA was based only on sounds heard or whether it was influenced by information from other aspects of the clinical evaluation, medical notes or prior knowledge. This study consisted of a control group comprising 10 healthy adults and a group with dysphagia comprising 14 adult patients. The sounds were recorded using a stethoscope placed on the lateral aspect of the thyroid cartilage while performing simultaneous videofluoroscopy. Boluses of 5 and 20mL of thin barium and 5mL of yogurt were offered. The sounds were recorded randomly on CDs, which were then sent for evaluation by speech therapists, who were asked to classify the swallowing as normal or abnormal and make qualitative comments. The comparison of the results of the acoustic analysis and the radiological images in relation to aspiration/penetration showed 66% specificity and 62% sensitivity. A significant correlation between individual reliability and an adequate rate of identification of swallowing disorders was observed. The interobserver reliability was low and varied. The results showed that 17 of the 20 sounds were identified, suggesting that swallowing sounds contained audible soundtracks, allowing classification. The authors concluded that there is a need to improve the accuracy of sound-detection techniques.

Researchers^(1)^ performed a study to compare the physiology of swallowing in dysphagic and healthy individuals and to evaluate the reliability of CA in the identification of dysphagia. The study included 14 dysphagic and 25 healthy adults. A stethoscope was positioned above the cricoid cartilage, and the subjects were offered 10mL of water. Deglutition was recorded using an audio recorder and compared to the videofluoroscopy. Two acoustic parameters were found that differentiated swallowing in healthy elderly and dysphagic individuals: the duration of the first burst tended to be higher in the elderly group compared with the dysphagia group, and the dysphagics required multiple swallowing attempts. A longer duration of swallowing apnea was observed in the elderly. The interobserver reliability coefficient for CA was 0.46. In relation to the presence of penetration/aspiration, when comparing the two tests, a specificity of 70% and a sensitivity of 94% were observed. The authors concluded that swallowing sounds contain clues that permit a reliable classification of swallowing.

Other researchers^(15)^ used acoustic recording techniques to analyze swallowing sound signals. Thirty healthy adults were evaluated while ingesting 10mL of barium and water. Fluoroscopy was carried out concurrently with the recording of the sounds using a microphone located on the trachea. For each recorded sound, the number of component sounds, the total duration of the sounds and the interval between each sound component were analyzed. The study allowed swallowing sounds to be broken down into three main components so that their durations could be quantified. The authors suggested that the results could be useful in the evaluation of variations in sounds in individuals with swallowing disorders.

One study^(16)^ investigated the most appropriate type of instrument to record swallowing sounds and the characteristics of the noise produced by these instruments. Two acoustic sensors were used: an accelerometer and a microphone. Deglutition of 10mL of water by 10 healthy adults was analyzed. Recordings were made with both instruments at the following different positions: midline between the thyroid and cricoid cartilages; the center of the cricoid cartilage; midline, below the cricoid cartilage; and the lateral border of the trachea below the cricoid cartilage. The results showed no significant differences between the different positions for either instrument. However, the microphone showed greater sensitivity.

Researchers^(17)^ conducted a study to identify the initial sounds of swallowing in adults and to compare them with the initial sound signals in babies to determine their stability. A group comprising 20 adults was assessed to compare their results with previous studies on low-risk preterm newborns using an accelerometer and microphone. Each participant was offered liquid, puree and solid. The rate of variance (RV) of the initial sound stability was compared between the groups. The adults' RV did not differ from the RV observed in infants aged >36 weeks for liquids; however, the adults' RV was lower than the RV displayed for infants aged <36 weeks. The authors concluded that the accelerometer and microphone were suitable methods to identify the initial sounds of swallowing.

## DISCUSSION

Deglutition sounds are produced due to biomechanical movements, *e.g.*, movements of bone structures, muscles, cartilage and mucous membranes before, during and after the passage of the bolus through the pharynx. CA is a method used to evaluate swallowing by detecting the sounds of these movements using amplification instruments.

The amplification instrument most used was the stethoscope^(3,6,8,15)^, but there was interest in the use of other instruments such as the accelerometer^(5,13,16,17)^ and microphone(5,10,11,15–17).

Several studies^(3,11,14)^ investigated the correlation between swallowing sounds and biomechanical events. Associations between a pre-click and the start of apnea, a pre-click and the start of epiglottis excursion, a click and the epiglottis returning to rest and a click and the end of the swallowing apnea have been suggested^(3)^. Another study^(14)^ noted that the opening sound of the upper sphincter was present in all swallowing.

Our research in the PubMed database was limited to studies conducted on adult humans and written in the English language. However, studies were also found that compared children with adults^(12,13)^. According to these studies, adult swallowing parameters differ from the parameters for children due to anatomical and physiological differences.

The highest number of publications belonged to the health area^(1,3,5–7,10,11,14–17)^, with CA being used most by speech therapists. Studies in bioengineering^(12,13)^ were also encountered, showing that the issue is current and challenging. These studies were conducted in an attempt to standardize the CA method, describe swallowing sound parameters and study various amplification instruments.

Most authors chose to offer intake of pasty^(3,5,10,12–14,17)^ and liquid^(1,3,5,10,12,16,17)^ consistencies. Intake of a solid consistency^(10,17)^ was offered less frequently due to the higher risk of penetration/aspiration.

The importance of this method in the clinical evaluation of swallowing, especially in speech therapy, was verified. This review showed that the CA method should be used in combination with other instruments (*e.g.*, pulse oximetry and clinical protocols), and its results can and should be considered parameters when evaluating swallowing.

## CONCLUSIONS

There has been interest in the cervical auscultation method from researchers not only in health areas but also in the field of bioengineering. It is an easily applied, low-cost method. Studies have sought to standardize swallowing sounds and modernize instruments and methodologies. The method of cervical auscultation is valid in clinical practice when used in conjunction with other instruments. Furth er research is needed to improve and validate this evaluation technique.
